# Observational and Reported Measures of Language and Pragmatics in Young People with Autism: A Comparison of Respondent Data and Gender Profiles

**DOI:** 10.1007/s10803-019-04288-3

**Published:** 2019-11-22

**Authors:** Alexandra Sturrock, Antonia Marsden, Catherine Adams, Jenny Freed

**Affiliations:** 1 School of Health Sciences, The University of Manchester, Manchester, UK; 2 Department of Human Communication, Development and Hearing, The University of Manchester, Ellen Wilkinson Building, Manchester, M13 9PL UK

**Keywords:** Language and communication, Gender, Autism spectrum disorder

## Abstract

Female children with autism spectrum disorder (FwASD) and performance intelligence quotient (PIQ) over 70 were compared with male children with ASD (MwASD) and typically developing (TD) controls (age 8–11 years) using a range of language and pragmatic measures. Functional ability was assessed using clinical observations and parent, teacher and self-reports. Results were compared between measures, and with direct assessments of language and pragmatics, in order to identify potential biases. This study found that FwASD performed better than MwASD but worse than TD controls on clinical observations of pragmatic ability. FwASD also performed worst overall on a parental measure of emotions. Additionally, there were patterns of differences between clinician, parent, teacher and self- reports and direct assessments, which indicate the need for assessment data to be collected from multiple informants. Findings also have implications for the accurate identification of ASD in females and appropriate provision of support.

**Electronic supplementary material:**

The online version of this article (10.1007/s10803-019-04288-3) contains supplementary material, which is available to authorized users.

## Introduction and Background

Females who meet the criteria for autism spectrum disorder (ASD) are at much greater risk than males of going undiagnosed (Dworzynski et al. 2012), or being diagnosed with other conditions (Giarelli et al. 2010). It is estimated that females represent a quarter of the total diagnosed population (Loomes et al. 2017) but may be particularly underrepresented in higher IQ groups (70 +; Nicholas et al. 2008). A contributing factor in this discrepancy seems to be the poorly understood presentation of surface behaviours associated with a female phenotype of ASD (Kreiser and White 2014). Studies have demonstrated fewer repetitive behaviours (Mandy et al. 2012), relationships with greater emotional reciprocity (Head et al. 2014), and better use of social communication compared with male counterparts (Park et al. 2012). In recent work, pragmatic and associated higher-level structural language skills have also been identified as areas of difference between the male and female phenotype of autism, using a range of direct assessments (Conlon et al. 2018; Kauschke et al. 2016; Sturrock et al. 2019). However, data in this area are complex, with differences between findings often appearing to be influenced by the person reporting on behaviours (clinicians, parents, teachers or the individual themselves), which may, in turn, be affected by the setting in which the child is being observed (e.g. at home or school; Mandy et al. 2012; Szatmari et al. 1994).

Higher-level linguistic ability is fundamentally linked to social competency (Durkin and Conti-Ramsden 2007) and pragmatic language development (Norbury and Bishop 2003) in the general population. Pragmatic language difficulties, e.g. comprehension of inference and discourse or impaired social use of language and social cognition (Adams et al. 2005), are thought to be some of the most prevalent features of ASD across groups of all verbal and cognitive abilities (Eigsti et al. 2011). Pragmatic and social skills are both linked to building peer relationships (Hebert-Myers et al. 2006) which are subsequently associated with well-being for this group (Mazurek 2013). They are also fundamentally associated with the core diagnostic domain of social communication (Baird and Norbury 2016). Although females with ASD (FwASD) are thought to outperform males with ASD (MwASD) on features of this domain, they still demonstrate deficits when compared with gender-matched controls (Head et al. 2014, Knickmeyer et al. 2008; Sturrock et al. 2019). With female relationships thought to be more dependent on collaborative discourse (Maccoby 2002), this may put FwASD at a particular disadvantage with their typically developing (TD) peer group. Pragmatic difficulties, like other features of autism, are commonly identified through observational and report measures, which may provide better insight into functional ability than direct assessments (Adams 2002). It is therefore expected that measures of pragmatics and functional use of language may contribute to the understanding of differences between FwASD and MwASD or gender-matched controls. In this study we will look particularly at clinical observation and parent, teacher and self- report measures.

### Report Measures for Females with ASD: Implications for Pragmatic and Language Assessment

The literature indicates that parents of children with ASD may report their child’s difficulties differently depending on gender (Holtmann et al. 2007; McLennan et al. 1993). For example, Holtmann et al. (2007) showed that parents rated their female child with ASD more critically than parents of a male child with ASD, despite no difference being identified by clinicians using an autism diagnostic schedule (ADOS, Lord et al. 2000). It was suggested that this could be the result of an interpretation bias by parents who may expect ‘more socially desired behaviour from their daughters than their sons’ (Holtmann et al. 2007, p. 361).

Szatmari et al. (1994) found differences between parent and teacher reports of autism severity for the same child, with teachers regularly reporting lower levels of difficulty than parents. Other studies indicate that teachers are likely to rate FwASD as having fewer observable difficulties in school than MwASD (Kamio et al. 2013; Mandy et al. 2012). Observable autism severity may be context-dependent (Posserud et al. 2006) and FwASD may particularly benefit from the structured environment of school, thus reducing externalising behaviours (Szatmari et al. 1994). Further; FwASD may also mask difficulties to a greater degree than MwASD in school because they perceive an increased need to fit in to social groups (Bargiela et al. 2016). These comparisons demonstrate difference in reporting of social communication difficulties and could therefore predict related differences in language and pragmatic abilities.

### Self-report Measures in ASD and Implications for Pragmatic and Language Assessment

Self-report is widely agreed to be essential in identifying difficulties which are not otherwise observable (Garcia and Gustavson 1997). In the ASD community, personal reflections from females have identified the experiences of late diagnosis (Bargiela et al. 2016) and camouflaging social difficulties (Hull et al. 2017). It is also essential to person-centred practice and as such is recommended in clinical guidelines (NICE 2012). The benefits and the limitations of self-report are subjectivity: providing rich representations of personal experiences as impacted upon by societal and psychological pressures. Data trends give us details of the shared experience by the group. However, objective accuracy may be affected by poor understanding of technical details, a lack of introspection, and/or reporting bias (Garcia and Gustavson 1997). Additionally, individuals with ASD may particularly struggle to self-assess accurately, due to underlying problems identifying social and emotional responses in others and the reflexive relationship this has with their own behaviour (Elmose 2016). Potential deficits in language and communication (Boucher 2012) and abstract reasoning (Solomon et al. 2011) experienced in ASD may also impact on the processing required for accurate self-reporting (Huang et al. 2017). It is unclear from existing literature whether these features would be experienced equally by females and males with ASD. Females with ASD are thought to have better reciprocity in relationships (Sedgewick et al. 2016) and potentially a more socially nurturing peer group (Gould and Ashton-Smith 2011) than males, which may support better self-awareness. Certainly, successful camouflaging commonly attributed to FwASD would seem to necessitate some degree of self-awareness and an ability to observe and copy acceptable social behaviours (Hull et al. 2017). However, in other work, FwASD were found to have increased tendencies towards self-deprecation than MwASD (Cohen et al. 2010). Lai et al. (2011) showed that FwASD were likely to report higher levels of social communication difficulties than evidenced by clinicians and attributed this to either better or more critical self-reflection than MwASD. These results could predict differences in related areas of self-report of pragmatic and language difficulties. More research is required to identify gender difference in self-reporting skills for ASD and, more generally, how children with ASD report pragmatic and language difficulties.

### Direct Assessment (DA)

Language and pragmatic abilities can be measured using direct assessment (DA), typically performed by a clinician in an isolated, one-to-one test environment. There is currently no comparison between language and pragmatic scores using DA and observation of functional ability. However, work in other areas may predict that the DA environment would be facilitative to children with ASD and therefore under-identify true functional difficulties in this area (Frith and Happé 1994). Test conditions favour the supposed cognitive preference of ASD groups for focus and attention to detail (Frith 1989), unlike functional environments, which require good multi-modal processing and shifting attention. Additionally, in DA settings the individual may recruit preferred skills of systemising/logicalising and pattern-finding (Baron-Cohen 2002, Lai et al. 2012) to interpret and answer socially-driven questions. The faster response times required in functional settings would likely render lengthy analytical approaches unworkable, again predicting better outcomes in DA. Furthermore, pragmatic DAs fundamentally change the contextually responsive nature of the paradigm under investigation (Adams 2002), potentially simplifying the task. It is possible that DAs provide important information about capacity in an optimal setting, but have less to contribute to assessment of function. It is likely that the ASD group will experience greater differences between functional report and DA outcomes on similar tasks, but it is not clear if there will be a gender effect.

In summary, pragmatics and higher level language skills as tested through DAs have demonstrated differences between females and males with ASD, as well as between FwASD and FwTD. It is likely that an observational assessment would identify similar differences and contribute to general understanding of the FwASD profile. Report measures from parents, teachers and individuals suggest discrepancies between how various reporters perceive strengths and difficulties for this group. This may be due to environmental and societal factors. Exploration of these discrepancies would provide a better informed, holistic picture of the individual and could help explain some of the inconsistencies in the literature. With pragmatic deficits (and by extension ASD symptomatology) dependent on these types of assessments, understanding their relative contribution could also contribute to clinical decision making during assessments.

Therefore, the aims of this study are as follows: (1) identify the pragmatic, language and social behaviours of FwASD compared with MwASD and matched TDs as observed using a clinical checklist and as reported by parents, children and teachers. We expect that detailed clinical observations of autism will distinguish between females and males with ASD. However, parent, teacher and child reports may not elicit gender differences. (2) Compare clinical observation measures with parent- and child-report measures. Here we expect observation to identify fewer difficulties for both ASD groups when compared with parent reports, and this difference may be greater for FwASD. It is not clear from the literature if we would expect FwASD to report greater or fewer difficulties compared with clinical observations. However, MwASD are expected to report fewer difficulties. (3) Compare parent and teacher ratings of social behaviours. We expect teachers to rate fewer difficulties than parents for the ASD group, and this difference may be greater for the FwASD group. (4) Compare functional measures with DAs. We expect the ASD group to perform worse on functional measures, but any effect of gender is unclear.

## Methods

### Participants

Thirteen females and thirteen males with ASD (8 years 11 months–11 years 6 months) were recruited through participating UK National Health Trusts, local charities and private educators. Inclusion criteria were: performance IQ (PIQ) over 70 on The Wechsler Abbreviated Scale of Intelligence (WAIS: Weschler 1999), evidence of multi-disciplinary ASD diagnostic assessment using the Diagnostic and Statistical Manual (APA 2013) or International Classification of Disease (WHO 1994) and scores above cut-off on the Autism Spectrum Screening Questionnaire (ASSQ: Ehlers et al. 1999). A TD group (*n* = 26) were matched on age and gender. They were recruited through The ESRC International Centre for Language and Communication Development (LuCiD) research group and database. These children fell below published cut-off scores using the ASSQ (Ehlers et al. 1999). All children had English as a first language, PIQ over 70 and no uncorrected hearing or visual impairment. Slight between-group variations in PIQ were controlled for in analysis. Screening assessments were administered by trained researchers during an initial visit. All parents (n = 52) completed questionnaires. All teachers were contacted, 47 teachers completed questionnaires (n = 11 females and n = 12 males with ASD and n = 12 females with TD and n = 13 males with TD).

### Inclusion Measures for Child Participants

*Wechsler Abbreviated Scale of Intelligence (WASI; Weschler*^1999^) Performance IQ obtained using two subtests for block design and matrix reasoning.

*The Autism Spectrum Screening Questionnaire (Ehlers et al.*^1999^*)* A 27-item screening tool designed to identify diagnostic features of autism, with high validity for participants with typical range IQ.

### Observational Checklists and Report Measures

*Pragmatic Rating Scale (PRS) (Landa*^2013^*; Landa et al. *1992*)* A 34-item checklist for pragmatic communicative behaviours, which provides total, overall and impairment scores as well as composite sub-scores of (1) speech acts/communicative intents, (2) presupposition and theory of mind, (3) discourse management, (4) speech and prosody behaviour, (5) supra-segmentals, and (6) non-verbal communication. Scores are out of 68 and higher scores indicate greater impairment. This measure was administered by the researchers (specialist autism clinicians) and scored in line with published guidelines (Landa 2011).

Visual observations of non-verbal communication meant blind scoring was not possible. Second-rater reliability was undertaken on 25% (12 cases) resulting in 81% percentage adjacent agreement (± 1) for total and composite scores. It was not genuinely possible for the second rater to be blind to gender, or effectively diagnosis, due to often clear differences in observable behaviour of the children. PRS scores were correlated with other assessment measures in order to ascertain accuracy in reporting, with moderate to strong positive correlations (reported in results).

In this study three subtests were chosen from the *Autism Diagnostic Observation Schedule Edition 2-Module 3 (ADOS*-*2; Lord et al.*^2012^*),* to elicit semi-structured conversation, semi-structured dialogue and narration. ADOS-2 is a diagnostic assessment designed to elicit communication, social interaction play and restricted, repetitive behaviours associated with autism. Researchers were trained in ADOS presentation and scoring prior to data collection. Items chosen: (1) *semi*-*structured conversation:* a conversation based around topics of interest to the child and researcher which are related to a given picture. (2) *semi*-*structured interview:* Answering questions about friends and relationships including describing a friend, and what is different between a friend and acquaintance. (3) *Narration:* telling a story from a wordless picture book.

*Child Communication Checklist-Second Edition (CCC-2) (Bishop, *2003*)* A 70-item questionnaire providing total as well as composite sub-scores for speech, syntax, semantics, coherence, inappropriate initiation, stereotyped language, use of context, non-verbal communication, social relations, and interests. Completed by parents. Scores are out of 150 and higher scores indicate greater impairment. Standard scores and percentiles are provided for each category, as well as overarching categories of General Communication and Social Interaction Deviance.

*Communication Checklist*-*Self Report (CC*-*SR) (Bishop et al.*^2009^*)* A 70-item questionnaire providing total and composite scores for language, pragmatics and social engagement. Scores are out of 150 and higher scores indicate greater impairment. It is designed and scored in a similar format to that outlined above for the CCC-2. Items are conceptually similar between the CC-SR and CCC-2. All children undertook the CC-SR. In each case questionnaires were presented to the child orally by the researcher and explanations provided if necessary. Raw scores were calculated for all children.

*Strengths and Difficulties Questionnaire-Parent Edition (SDQ*-*P) (Goodman,*^1997^*)* A 25-item behaviour questionnaire providing total as well as composite scores of pro-social behaviour, peer relations, conduct problems, emotional regularity, hyperactivity, internalising and externalising behaviours and impact for the child. Statements like ‘considerate of other people’s feelings’ are graded as: (0) not true, (1) somewhat true, (2) certainly true. Each answer is scored 0-2. Scores are out of 50 and higher scores indicate greater impairment. They were calculated using published guidelines.

*Strengths and Difficulties Questionnaire-Teacher Edition (SDQ*-*T) (Goodman,*^1997^*)* A 25-item behaviour questionnaire following the same format as described in the parent version above.

### Direct Assessment (DA)

*Pragmatics: Local Coherence Inference task (Joliffe and Baron*-*Cohen*^1999^*)* An 18-item experimental measure, testing understanding of inferred meaning which provides coherence to a short story. The child reads the story, which purposely omits an overt bridging reference between an initiating event and a consequence. The child is asked to correctly identify the missing information from a choice of three, all of which could be appropriate, but one constitutes the best fit. Responses are scored correct/incorrect. Lower scores indicate greater impairment. Full details about the measure and adaptations are included in the Online Appendix 1.

*Pragmatics: Figurative Language Task (MacKay and Shaw*^2004^*)* An experimental measure of 21 items testing understanding of figurative language (irony, hyperbole, metonym, indirect comment, rhetorical question, understatement and metaphor). The participants are presented with an example of figurative language and a picture which provides contextual information to support accurate interpretation. The child is given one point for describing the true meaning of the figurative language and another for determining speaker intention. Lower scores indicate greater impairment. Full details about the measure and adaptations are included in the Online Appendix: 2.

*British Picture Vocabulary Scale (BPVS*-*3) (Dunn et al.*^1997^*)* The child demonstrates receptive word knowledge by identifying a target word from a choice of four pictures following spoken presentation of the word target by the assessor. Scores are out of 132, lower scores indicate greater impairment.

*The Clinical Evaluation of Language Fundamentals-Fourth Edition (CELF-4)* Recalling Sentences subtest (Semel et al. 2006*)* The child is presented with a spoken sentence and is asked to recite this verbatim. Errors made by the child are tallied to produce a raw and standardised score. Scores are out of 95, lower scores indicate greater impairment.

*The Clinical Evaluation of Language Fundamentals-Fourth Edition (CELF*-*4): Word Associations Subtest (Semel et al.*^2006^*)* The child is asked to generate words within super-ordinate categories of animals, food, and occupations, following the instruction: “Name different jobs or occupations that people might have. Name as many as you can in 1 min. For example, you could say *babysitter* or *mechanic*. Now you name some more. Start now.” Raw scores are generated for each category. Lower scores indicate greater impairment.

### Procedure

Children were seen individually at their home or school over three sessions of 60 min, with parents in attendance for at least the first visit. In this initial meeting children were video recorded undertaking specific communicative tasks based on ADOS-2 (Lord et al. 2012). Discourse samples were subsequently rated by researchers using PRS (Landa 2013; Landa et al. 1992). In the initial visit parents completed The CCC-2 (Bishop 2003), children completed The CC-SR (Bishop et al. 2009) and parents also completed The SDQ-P (Goodman 1997). Any questions could be directed to the researcher during the session. Subsequent sessions were primarily for collecting language and pragmatic DAs, although the self-report questionnaire was sometimes presented in smaller chunks, e.g. 20 questions per visit over 3 visits to support children with lower attention levels. This was available for all children but only required for the ASD groups. Level of attention and fatigue was determined by the researcher, based on observation and quality of answers. Teachers were given a copy of The SDQ-T (Goodman 1997) directly by the researcher or via the parent. Details for completing the questionnaire were included in the document but teachers could contact the researcher with any questions. The completed questionnaire was collected directly from the teacher or posted to The University of Manchester. The procedure for all established questionnaires and direct language/discourse measures were derived directly from published guidelines. Any adaptations are detailed in the Online Appendix.

## Results

Clinician observations using the PRS and parent reports on both the CCC-2 and SDQ(P) were obtained for all children. 51 children completed the CC-SR (1 × FwASD missing). 48 teachers completed the SDQ(T) (2 × FwASD, 1 × MwASD and 1 × FwTD were missing). Direct assessment typically included the full cohort of 52 children, although any discrepancies are noted in the relevant table. Table 1 shows descriptive statistics for age, PIQ and autism severity (using ASSQ ratings) for ASD and TD groups. Group differences were analysed using separate 2 (Gender) × 2 (Group) ANOVAs. The groups were well-matched for chronological age: Group [*F*(1,48) = 2.924, *p* = .094,, ŋ^2^ = .057]; Gender [*F*(1, 48) = 1.634, *p* = .207, ŋ^2^ = .033]; Group × Gender interaction [*F*(1,48) = 1.898, *p* = .175, ŋ^2^ = .038]. There was a small but significant main effect of Group on PIQ [*F*(1,48) = 0.072, *p* = .021, ŋ^2^ = 0.105] with the TD group showing marginally higher PIQ (Mean = 116.62) than the ASD group (Mean = 107.08). There were no other significant effects on the PIQ measure [Gender: *F*(1,48) = 0.072, *p* = .790, ŋ^2^ = .001; Group × Gender interaction: *F*(1,48) = 0.001, *p* = .970, ŋ^2^ = .000]. Between group analyses were corrected for PIQ. There was a significant effect of Group on autism severity ratings [*F*(1,46) = 257.966, *p* = − .001, ŋ^2^ = 0.849] with TDs showing lower scores, i.e. fewer difficulties, on the ASSQ (Mean = 2.15) than the ASD group (Mean = 33.36). There were no other significant effects on the ASSQ scores [Gender: *F*(1,46) = 0.360, *p* = .551, ŋ^2^ = .008; Group × Gender interaction: *F*(1,46) = 0.043, *p* = .836, ŋ^2^ = .043]. As this was an expected group difference and did not directly impact on analysis of gender difference. It was not introduced as a covariate in subsequent analysis, because severity of autistic symptomatology (in terms of language and communication) was a key factor under investigation. During early stages of data analysis it was noted that not all variables were normally distributed. However, sample measures were investigated using parametric and non-parametric analysis, and significance values remained consistent. The decision was made to use parametric analysis to allow detailed interaction analysis to be undertaken. Analyses were conducted at the 5% significance level. Bonferroni adjustment for multiple testing was not performed as the statistical tests were correlated. More weight should be given to the primary, rather than secondary (subsection) analyses. Table 1

**Table 1:** Descriptive statistics for chronological age (in months) and PIQ scores for ASD and TD groups by gender

	ASD (*n* = 26) Mean (*SD*)	TD (*n* = 26) Mean (*SD*)	Gender overall Mean (*SD*)
PIQ (raw score)			
Female	107.69 (17.32)	117.08 (14.95)	112.38 (16.56)
Male	106.46 (11.93)	116.15 (13.10)	111.31 (13.23)
Group overall	107.08 (14.59)	116.62 (13.78)	111.85 (14.85)
Age (in months)			
Female	124.46 (8.35)	125.23 (6.98)	124.85 (7.55)
Male	118.31 (9.93)	125.46 (7.88)	121.88 (9.51)
Group overall	121.39 (9.52)	125.35 (7.29)	123.37 (8.63)
Autism severity (ASSQ: max score 54)			
Female	32.83 (8.83)	1.77 (2.77)	16.68 (17.04)
Male	34.42 (9.93)	2.54 (3.76)	17.84 (17.79)
Group overall	33.63 (9.23)	2.15 (3.26)	17.26 (17.25)

### Between Group Comparisons

Primary analysis was conducted on total scores, using the PRS, CCC-2, CC-SR, SDQ-P and SDQ-T, and results are reported in this order. Raw scores were analysed using a series of 2 (Group) × 2 (Gender) ANCOVAs with PIQ covariate. Descriptive statistics [means (M) and standard deviations (SD)] of total scores are shown in Table 2. Subsections from the various measures were analysed in secondary analysis. Tables and details of subsection comparisons are found in the Online Appendices (3–7) with key findings summarised in the main text.

**Table 2:** Descriptive statistics for total scores on the PRS, CCC-2, CC-SR, SDQ-T, SDQ-T: 2 × groups by 2 × gender ANCOVA (PIQ covariate)

	ASD (n = 26) Mean (SD)	TD (n = 25) Mean (SD)	Gender overall Mean (SD)	Results of ANOVA		
	Raw score	Raw score		Group	Gender	Interaction
PRS total score (max score 68)				*p* <.001	*p* = .002	*p* = .009
Female	11.38 (8.92)	1.75 (1.96)	6.76 (8.11)			
Male	21.69 (8.03)	2.69 (2.69)	12.19 (11.32)			
Group overall	16.54 (9.84)	2.24 (2.37)	9.53 (10.16)			
CCC2 total score (max score 150)				*p* <.001	*p* = .802	*p* = .987
Female	105.92 (34.04)	10.09 (10.73)	62.00 (55.08)			
Male	104.92 (19.36)	14.62 (21.18)	59.77 (50.16)			
Group overall	105.42 (27.13)	12.54 (17.01)	60.84 (52.05)			
CC-SR total score (max score 150)				*p* = .005	*p* = .351	*p* = .645
Female	62.64 (25.87)	37.77 (18.05)	49.17 (24.92)			
Male	66.18 (22.92)	46.69 (20.69)	56.43 (23.58)			
Group overall	64.55 (23.84)	42.23 (19.56)	52.95 (24.27)			
SDQ-P Total score (max score 50)				*p* <.001	*p* = .484	*p* = .300
Female	25.46 (5.74)	4.69 (4.63)	15.08 (11.76)			
Male	23.31 (4.03)	5.23 (4.92)	14.27 (10.22)			
Group overall	24.38 (4.98)	4.96 (4.69)	14.67 (10.91)			

### Pragmatic Rating Scale

*Total:* There was a small but significant main effect of Gender [F(1,47) = 10.354, *p* = .002, ŋ^2^ = 0.181], Group [F(1, 47) = 56.300, *p* < .001, ŋ^2^ = .545] and Group × Gender interaction [F(1, 47) = 7.402 *p* = .009, ŋ^2^ = .136]. Females (overall) were rated as having fewer difficulties (Mean = 6.58) than males (overall) (Mean = 12.19) and the TD group (Mean = 2.23) had fewer difficulties than the ASD group (Mean = 16.54). MwASD were more impaired than all other groups and this effect reached significance.

*Subsections (Online Appendix 3)* There were significant group differences on all subsections. There were significant gender and interaction effects on impact and composite scores for discourse management and speech and language behaviours. There were significant gender, but not interaction, effects overall and for non-verbal behaviour. FwASD performed better than MwASD, but behind FwTD, on all subsection analysis of the PRS. There was no significant effect of gender on speech acts and communicative intent.

### Childhood Communication Checklist

*Total* There was a medium but significant main effect of Group [*F*(1, 45) = 177.59, *p* <.001, *ŋ*^*2*^ = .798], but not Gender [*F*(1,45) = .064, *p* =.802, *ŋ*^*2*^ = .001] nor Group × Gender interaction [*F*(1, 45) = .017, *p* = .897, *ŋ*^*2*^ = .000]. The TD group were rated as having fewer difficulties (Mean = 12.54) than the ASD group (Mean = 105.42).

*Subsection (Online Appendix 4)* There were group but not gender nor interaction effects reaching significance throughout. Parents rated FwASD as performing worst on several composite items (speech, syntax, semantics and non-verbal abilities). They rated MwASD worst on general communication, coherence, inappropriate initiation, stereotypies (repetitive behaviours), contextual use of language, social skills and interests.

### Communication Checklist-Self report

*Total* There was a small but significant main effect of Group [F(1, 45) = 8.79, *p* = .005, ŋ^2^ = .163], but not Gender [F(1,45) = .889, *p* =.351, ŋ^2^ = .019] nor Group × Gender interaction [F(1, 45) = .216, *p* = .645, ŋ^2^ = .005]. The TD group were rated as having fewer difficulties (Mean = 42.23) than the ASD group (Mean = 64.55).

*Subsection (Online Appendix 5)* There were group but not gender nor interaction effects reaching significance across all subsections: language, pragmatics and social communication.

### Strengths and Difficulties Questionnaire (Parent Report)

*Total* There was a medium but significant main effect of Group [*F*(1, 47) = 180.193, *p* < .001, *ŋ*^*2*^ = .793], but not Gender [*F*(1,47) = .497, *p* = .484, *ŋ*^*2*^ = .010] nor Group × Gender interaction [*F*(1, 47) = 1.097, *p* = .300, *ŋ*^*2*^ = .023]. The TD group were rated as having fewer difficulties (Mean = 4.96) than the ASD group (Mean = 24.38).

*Subsection (Online Appendix 6)* There were group differences across all items, with TDs performing better than ASDs. Parents rated MwASD to have most difficulties in peer relations, hyperactivity and impact and FwASD to have most difficulties in pro-social behaviour, emotions, conduct, internalising and externalising behaviours. However, there was only a significant effect of gender on the composite score of emotions [*F*(1,47) = 7.172, *p* = .010, *ŋ*^*2*^ = .132], with FwASD (Mean = 8.38), performing worse than all other groups: MwASD (Mean = 6.31), FwTD (Mean = 1.31), MwTD (Mean = .69).

### Strengths and Difficulties Questionnaire (Teacher Report)

*Total* There was a small but significant main effect of Group [*F*(1, 42) = 31.142, *p* < .001, *ŋ*^*2*^ = .426], but not Gender [*F*(1,42) = 2.007, *p* = .164, *ŋ*^*2*^ = .046) nor Group × Gender interaction [*F*(1, 42) = 1.059, *p* = .309, *ŋ*^*2*^ = .025]. The TD group were rated as having fewer difficulties (Mean = 3.12) than the ASD group (Mean = 13.34).

*Subsection (Online Appendix 7)* Group but not gender nor interaction effects reached significance according to teachers across all subsection items. ASDs performed worse than TDs and MwASD were generally considered to perform worst of all. Teachers identified FwASD as performing lowest on composite scores of emotions and internalising behaviours.

### Summary

The PRS showed significant gender and group differences for total scores and on a range of composite subsections. Parents, the children themselves and the teachers identified a similar level of impairment for both gender groups when compared with TD controls. Overall clinicians, parents, and teachers tended to score MwASD with more difficulties than FwASD, although this did not always reach significance. However, teachers and parents both reported elevated difficulties for FwASD in key areas of emotions and internalising behaviours. This reached significance for the parent rating of emotional difficulties.

### Between Measure Comparisons: (Observations and Reports)

Total raw scores were compared between the following measures: (1) clinician (PRS) and parent (CCC-2), (2) clinician (PRS) and child (CC-SR), (3) Parent (CCC-2) and child (CC-SR, (4) Parent (SDQ-P) and teacher (SDQ-T).

A correlation was conducted for each paired comparison, to identify overall agreement. Further analysis was used to calculate effect of gender, group nor interaction. Firstly, a new variable was calculated to represent the difference (D) in scores between the two measures. Where total scores were directly comparable (e.g. for the child/parent and teacher/parent comparisons) this variable was calculated: D = (measure1–measure2), e.g. D = (CCSR–CCC2). Where test items were not directly comparable (e.g. for the PRS clinician and CCC-2 parent scores) a preliminary stage converted raw scores to standardised scores (z scores with a mean of 0 and SD of 1), then D = (SS1–SS2), e.g. D = (SSPRS–SSCCC2). Finally 2 (Gender) × 2 (Group) ANOVA analysis was conducted using the resulting variable.

Table 3 shows descriptive statistics for raw and standardised scores (mean and SD) as well as significance values for the 2 (Group) × 2 (Gender) ANOVA analysis. Correlations are described in the text.

**Table 3:** Descriptive statistics for total scores for PRS, CCC-2, CC-SR and SDQ(P) and SDQ(T) significance of difference between measures: 2 × group × 2 gender ANOVA

Comparison items	Mean RS (SD) Mean SS (SS-SD) PRS	Mean RS (SD) Mean SS (SS-SD) CCC-2	Significance
PRS total–CCC2 total			
FwTD (n:11)	1.77 (1.88) − .75 (.19)	10.09 (10.72) − .97 (.21)	Group: *p* = .030
MwTD (n:13)	2.69 (2.68) − .66 (.27)	14.61 (21.18) − .88 (.41)	
FwASD (n:13)	11.38 (8.82) .20 (.88)	105.92 (34.04) .87 (.65)	Gender: *p* = .003
MwASD (n:13)	21.69 (8.03) 1.21 (.79)	104.92 (19.36) .85 (.37)	
			Interaction: *p* = .003

	PRS	CC-SR	
PRS total–CC-SR total			
FwTD (n:13)	1.77 (1.88) − .75 (.19)	37.77 (18.05) − .63 (.74)	Group: *p* = .124
MwTD (n:13)	2.69 (2.68) − .66 (.27)	46. 69 (20.69) − .26 (.85)	
FwASD (n:11)	11.38 (8.82) .20 (.88)	62.64 (25.87) .40 (1.07)	Gender: *p* = .274
MwASD (n:13)	21.69 (8.03) 1.21 (.79)	66.18 (22.92) .55 (.94)	
			Interaction: *p* = .048

	CCC-2	CC-SR	
CCC2 total–CC-SR total			
FwTD (n:11)	10.09 (10.72)	37.77 (18.05)	Group: *p* <.001
MwTD (n:13)	14.61 (21.18)	46. 69 (20.69)	
FwASD (n:11)	105.92 (34.04)	62.64 (25.87)	Gender: *p* = .654
MwASD (n:13)	104.92 (19.36)	66.18 (22.92)	
			Interaction: *p* = .777

	SDQ(P)	SDQ(T)	
SDQ(P) total–SDQ(T) total			
FwTD (n:12)	4.69 (4.63)	2.75 (3.02)	Group: *p* < .001
MwTD (n:13)	5.23 (4.92)	3.46 (5.14)	
FwASD (n:11)	25.46 (5.74)	11.50 7.61	Gender: *p *= .142
MwASD (n:13)	23.31 (4.03)	15.18 (6.24)	
			Interaction *p* = .047

**Fig. 1:**
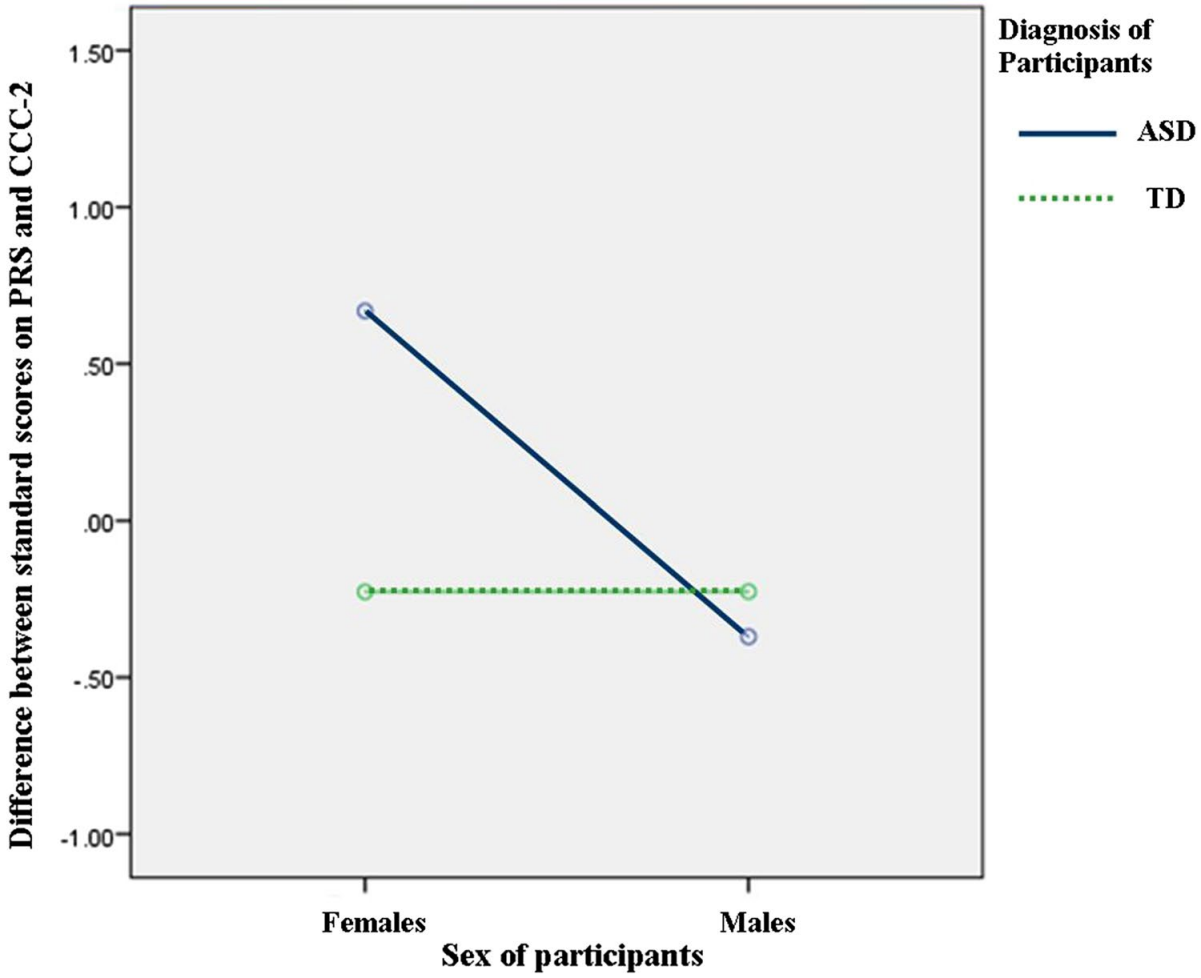
Graph showing ANOVA (2 gender × 2 group) on variable of difference between clinician (PRS) and parent (CCC-2) reports of pragmatic and language abilities

*PRS (Clinician) Total/CCC*-*2 (Parent) Total* There was a moderate positive correlation (Cohen 1992) between the PRS (Mean = 9.38, SS = 10.11) and CCC-2 (Mean = 60.84, SS = 52.05), n:50, *r* = .749, *p *< .001. There was a significantly bigger difference between clinician and parent scores for the ASD group when compared with the TD group [*F*(1, 46) = 5.034, *p* = .030, *ŋ*^*2*^ = .099]. There was also a significantly bigger difference between clinician and parent scores for females when compared with males [*F*(1, 46) = 9.611, *p* = .003, *ŋ*^*2*^ = .173] and there was an interaction effect [*F*(1, 46) = 9.629, *p* = .003, *ŋ*^*2*^ = .173]. Figure 1 shows parents identified more difficulties for FwASD (Mean = 105.92, SS = 34.04) than clinicians (Mean = 11.38, SS = .20) and marginally fewer difficulties for MwASD (Mean = 104.92, SS = .85) than clinicians (Mean = 21.69, SS = 1.21). TD parents and clinician scores were most closely matched.

**Fig. 2:**
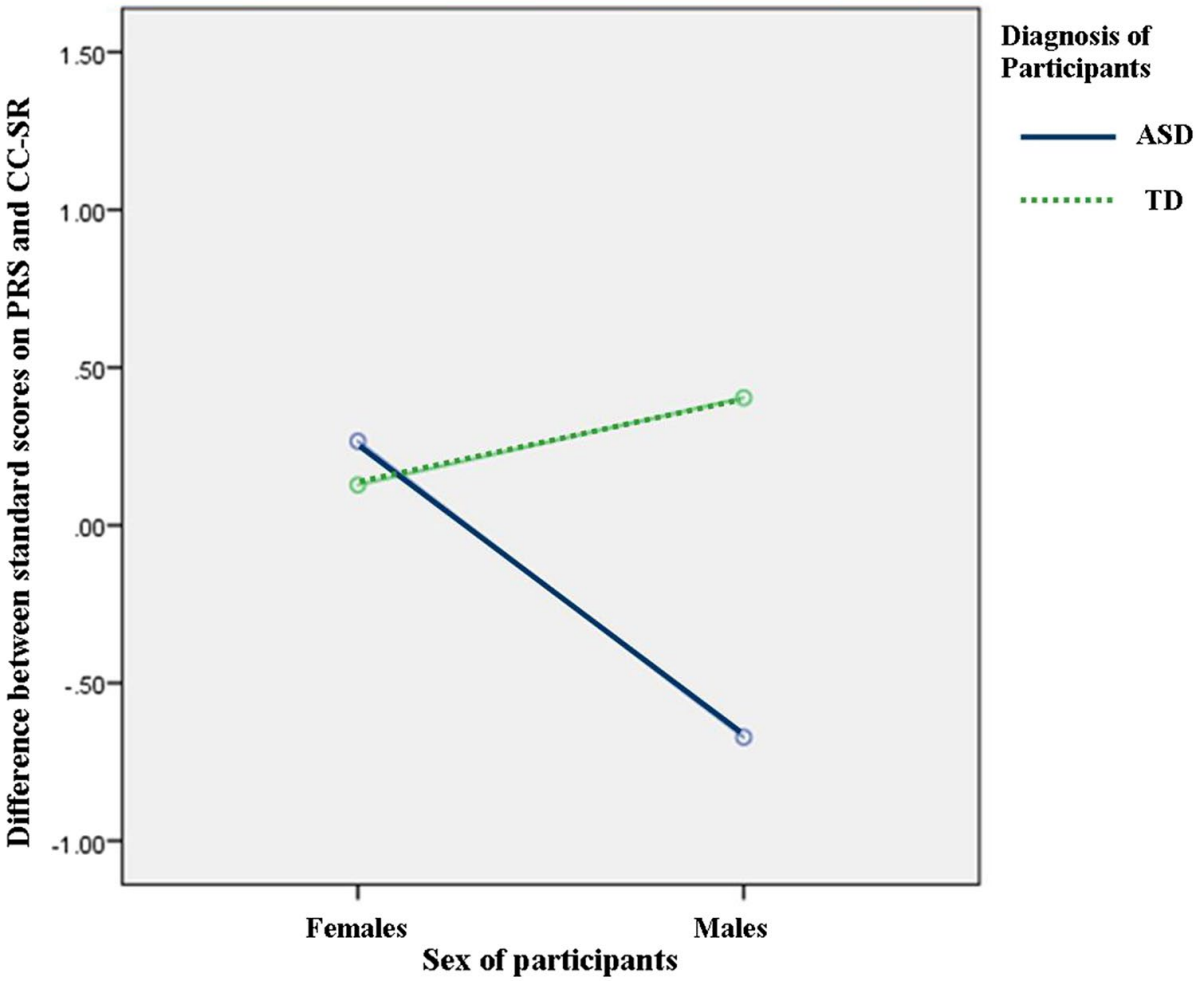
Graph showing ANOVA (2 gender × 2 group) on variable of difference between clinician (PRS) and child (CC-SR) reports of pragmatic and language abilities

*PRS (Clinician) Total/CC*-*SR (Child) Total* There was a moderate positive correlation between the PRS (Mean = 9.38, SS = 10.11) and CC-SR (Mean = 52.95, SS = 24.27), n:50, *r* = .388, *p *< .005. There was no significant difference between clinicians and children scores for the ASD group when compared with the TD group [*F*(1, 46) = 2.453, *p* = .124, *ŋ*^*2*^ = .051]. There was also no significant difference between clinicians and children scores for females when compared with males [*F*(1, 46) = 1.224, *p* = .274, *ŋ*^*2*^ = .026]. However, there was a small interaction effect [*F*(1, 46) = 4.122, *p* = .048, *ŋ*^*2*^ = .082). Figure 2 shows that MwASD rated fewer difficulties (Mean = 66.18, SS = .55) than clinicians (Mean = 21.69, SS = 1.21). A score of .00 indicates no differences between scores according to different reporters. FwASD, FwTD and MwTD were closer to clinician scores then MwASD and FwASD (Mean = 62.64, SS = .40), FwTD (Mean = 37.77, SS = -.63) and MwTD (Mean = 46.69, SS = − .26) all reported marginally more difficulties than those observed by the clinician: FwASD (Mean = 11.38, SS = .20), FwTD (Mean = 1.77, SS = − .75), MwTD (Mean = 2.69, SS = -.66).

**Fig. 3:**
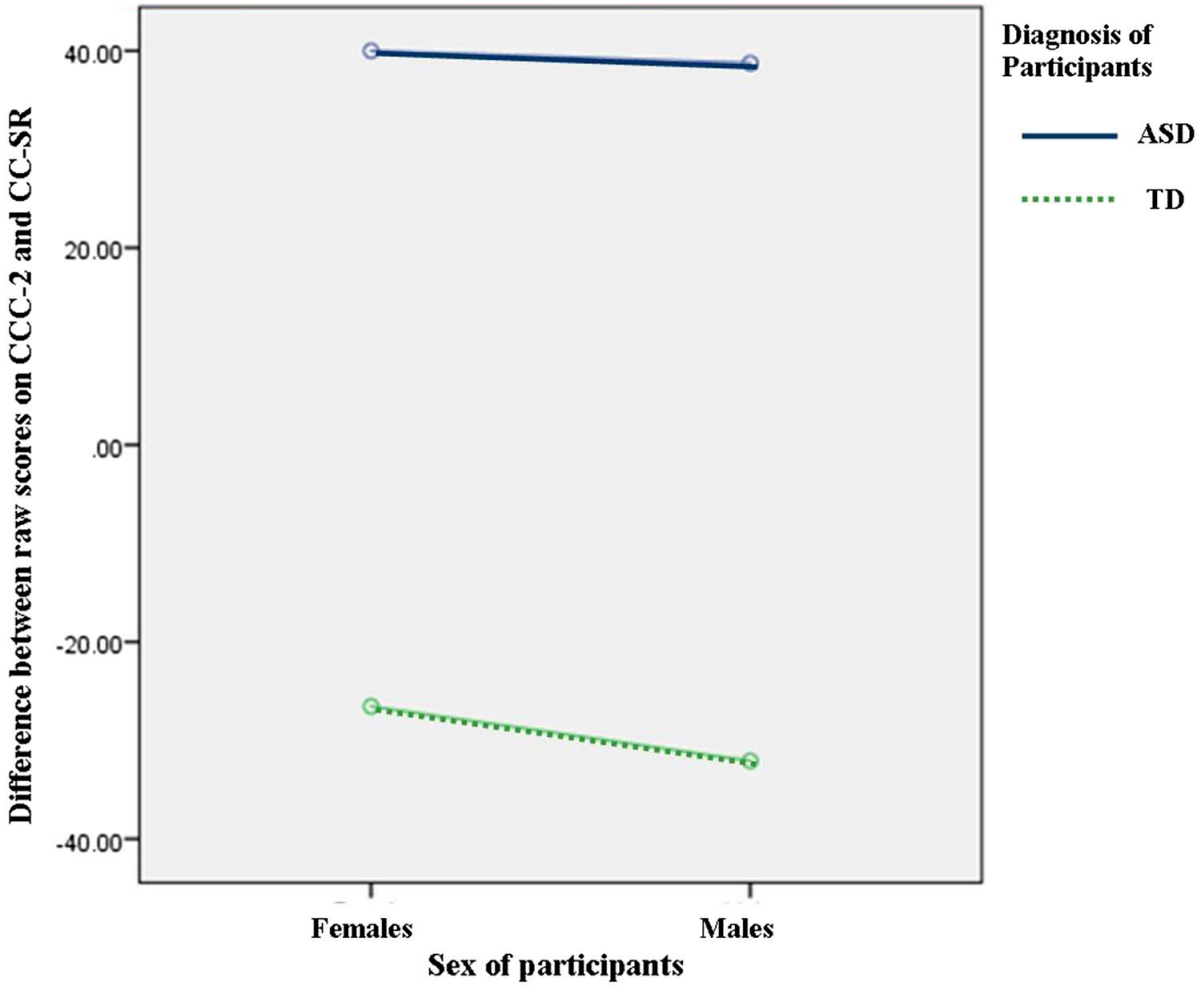
Graph showing ANOVA (2 gender × 2 group) on variable of difference between parent (CCC-2) and child (CC-SR) reports of pragmatic and language abilities

*CCC*-*2 (Parent) Total/CC*-*SR (Child) Total* There was a moderate positive correlation between the CCC-2 (Mean = 60.38) and CC-SR (Mean = 52.95), n:50, *r*  =  .555, *p *< .001. There was significantly bigger difference between parent and child scores for the ASD group when compared with the TD group [*F*(1, 44) = 83.380, *p* < .001, *ŋ*^*2*^ = .655]. There was no significant difference between parents and children scores when comparing females to males [*F*(1, 44) = .203, *p* = .654, *ŋ*^*2*^ = .005] and there was no interaction effect [*F*(1, 44) = .081, *p* = .777, *ŋ*^*2*^ = .002]. Figure 3 shows that FwTD (Mean = 37.77) and MwTD (Mean = 46.69) both reported slightly more difficulties than those observed by the parents: FwTD (Mean = 10.09), MwTD (Mean = 14.61). However, both ASD groups of children reported fewer difficulties than their parents: FwASD child (Mean = 62.64) versus parent (Mean = 105.92) and MwASD child (Mean = 66.18) versus parent rating (Mean = 104.92).

**Fig. 4:**
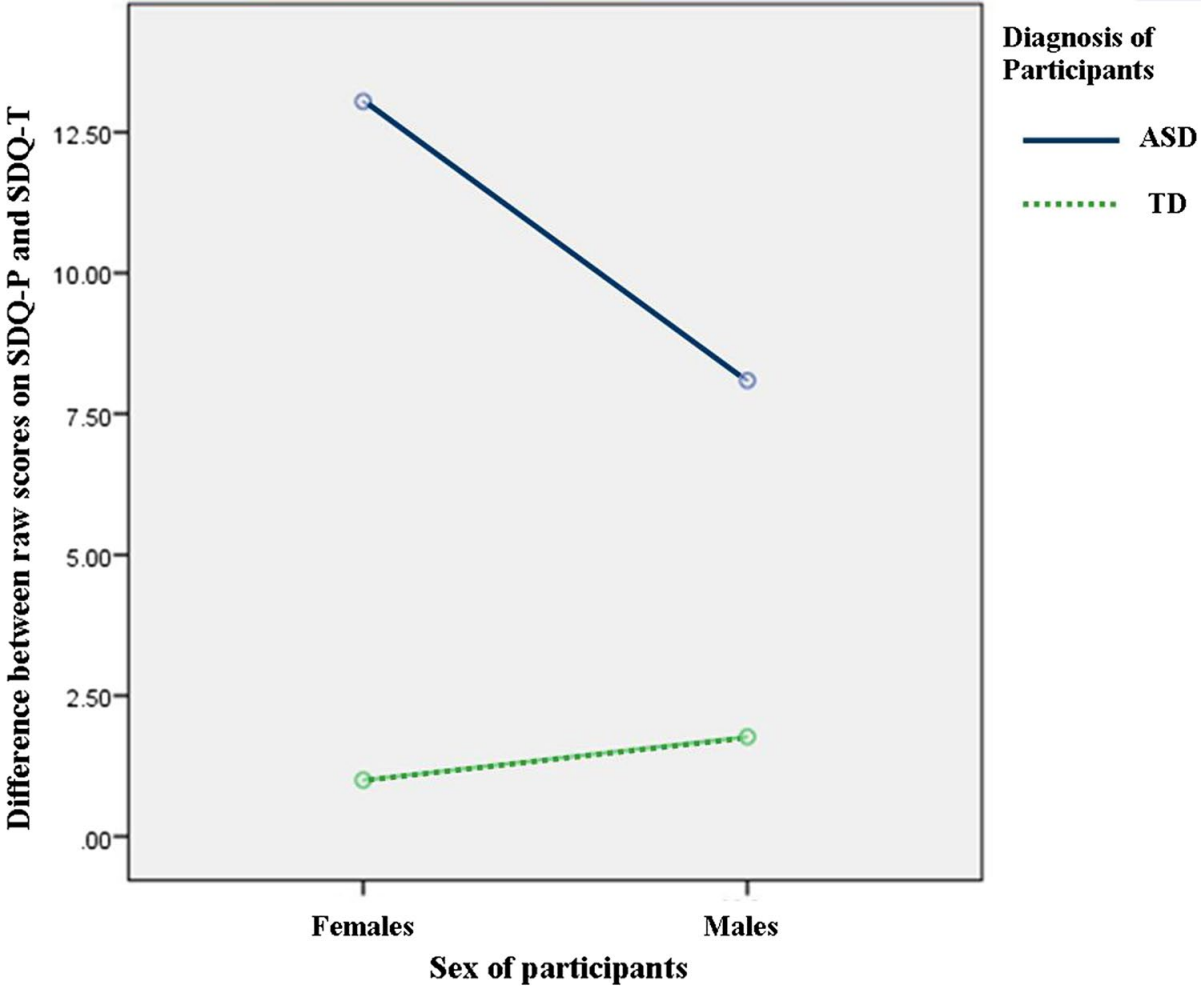
Graph showing ANOVA (2 gender × 2 group) on variable of difference between parent (SDQ_P) and teacher (SDQ-T) reports of behaviour

*Total Score SDQ*-*Parent/SDQ*-*Teacher* There was a moderate positive correlation between the SDQ(P) (Mean = 14.67/SD10.91) and SDQ(T) (Mean = 7.90/SD7.64), n:47, *r* = .781, *p *< .001. Relative to TDs, parents score children with ASD with significantly more difficulties than teachers [*F*(1, 43) = 43.120, *p* < .001, *ŋ*^*2*^ = .501]. Relative to males there was no significant differences in how parents and teachers score females compared with males [*F*(1, 43) = 2.239, *p* = .142, *ŋ*^*2*^ = .049]. However, there was an interaction effect [*F*(1, 43) = 4.188, *p* = .047, *ŋ*^*2*^ = .089]. Figure 4 shows that FwASD were scored as having more difficulties by parents (Mean = 25.46) when compared with teacher scores (Mean =11.50). This effect was also greater than the difference found between the MwASD parent (Mean =23.31) and teacher scores (Mean =15.18).

### Summary

Overall there was either small or moderate correlation between the different measures in this analysis, when all participants were grouped together. However, there were various significant differences identified between measures. Parents identified more difficulties for FwASD and marginally fewer difficulties for MwASD when compared with clinicians. In self- report, FwASD, FwTD and MwTD rated themselves as having slightly more difficulties than those identified by the clinicians. However, MwASD identified far fewer. In the ASD group, parents typically scored their child with more difficulties than the children themselves. However, TD children typically rated themselves as having more difficulties than *their* parents did. Parents of children with ASD consistently reported greater difficulties than teachers. But this discrepancy was significantly greater between parent and teachers scores for FwASD.

### Between Measure Comparison of Language and Pragmatics (Functional vs. DA)

Comparisons were made between direct assessments (DA) and either the clinician observations (PRS) or the parent questionnaire (CCC-2). Items for comparison were chosen because of conceptual similarity between the pragmatic/language features being assessed either by observation, report or DA. They are detailed in Table 4.

**Table 4:**
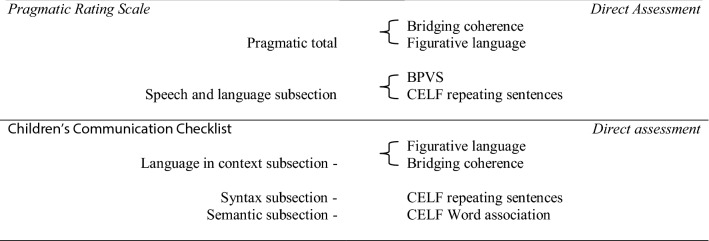
Comparisons conducted between direct language and pragmatic assessments with either the PRS or the CCC-2

Again a correlation was conducted for each paired comparison and a 2 (Gender) × 2 (Group) ANOVA analysis was conducted on difference (D) between measures. D was calculated in the same way as detailed in (2), e.g. raw score was converted to standardised scores (z scores with mean of 0 and SD of 1), then variable D was calculated: D = (SS1–SS2), e.g. D = (SSPRS–SSCCC2). Table 5 shows overall correlation between measures, descriptive statistics for raw and standardised scores (mean and SD) as well as significance values for the 2 (Group) × 2 (Gender) ANOVA analysis.

**Table 5:** Descriptive statistics for observational and report measures (PRS and CCC-2) and Direct assessment (DA), correlations and significance of difference between measures: 2 × group × 2 gender ANOVA

Comparison items	Mean RS (SD) Mean SS (SS–SD) PRS	Mean RS (SD) Mean SS (SS–SD) DA	Significance
PRS total (max score 68)–bridging coherence (max score 18)			
All participants (n:50) correlation	9.38 (10.11)	14.73 (2.41)	*p *< .001 r = − .510
FwTD (n:13)	1.77 (1.88) − .75 (.19)	16.08 (1.19) − .56 (.49)	Group: *p* <.001
MwTD (n:13)	2.69 (2.68) − .66 (.27)	15.54 (1.94) − .34 (.80)	
FwASD (n:13)	11.38 (8.82) .20 (.88)	14.62 (2.63) .048 (1.09)	Gender: *p* = .002
MwASD (n:13)	21.69 (8.03) 1.21 (.79)	12.69 (2.32) .85 (.96)	
			Interaction: *p* = .022
PRS total (max score 68)–figurative language (max score 21)			
All participants (n:50) correlation	9.38 (10.11)	16.85 (3.14)	*p *< .001 r = − .504
FwTD (n:13)	1.77 (1.88) − .75 (.19)	19.38 (.97) − .81 (.28)	Group: *p* = .635
MwTD (n:13)	2.69 (2.68) − .66 (.27)	17.54 (2.76) − .22 (.88)	
FwASD (n:13)	11.38 (8.82) .20 (.88)	16.46 (2.33) .12 (.74)	Gender: *p* = .165
MwASD (n:13)	21.69 (8.03) 1.21 (.79)	14.00 (3.42) .91 (.74)	
			Interaction: *p* = .187
PRS speech and language (max score 8)–BPVS (max score 132)			
All participants (n:50) correlation	1.04 (1.55)	109.69 (13.01)	*p *= .007 r = − .372
FwTD (n:13)	.08 (.28) − .62 (.18)	111.31 (5.63) − .12 (.43)	Group: *p* = .051
MwTD (n:13)	.15 (.38) − .57 (.24)	116.08 (7.70) − .49 (.59)	
FwASD (n:13)	1.08 (.76) .025 (4.91)	106.69 (16.34) .23 (1.26)	Gender: *p* = .019
MwASD (n:13)	2.85 (1.99) 1.17 (1.29)	104.69 (16.65) .38 (1.28)	
			Interaction: *p* = .326
PRS speech and language (max score 8)–CELF recalling sentences (max score 95)			
All participants (n:50) correlation	1.04 (1.55)	69.00 (12.71)	*p *= .001 r = − .447
FwTD (n:13)	.08 (.28) − .62 (.18)	72.23 (12.71) − .25 (1.00)	Group: *p* = .220
MwTD (n:13)	.15 (.38) − .57 (.24)	74.23 (10.83) − .41 (.85)	
FwASD (n:13)	1.08 (.76) .025 (4.91)	68.85 (10.13) .01 (.80)	Gender: *p* = .070
MwASD (n:13)	2.85 (1.99) 1.17 (1.29)	60.69 (13.76) .65 (1.09)	
			Interaction: *p* = .607

	CCC2	DA	
CCC2 language in context (max score 15)–bridging coherence (max score 18)			
All participants (n:50) correlation	7.76 (7.12)	14.73 (2.41)	*p *< .001 r = − .525
FwTD (n:11)	.64 (1.29) − 1.00 (.18)	16.08 (1.19) .56 (.49)	Group: *p* = .002
MwTD (n:13)	2.08 (3.57) − .80 (.50)	15.54 (1.94) .34 (.80)	
FwASD (n:13)	12.92 (4.50) .72 (.63)	14.62 (2.63) − .048 (1.09)	Gender: *p* = .223
MwASD (n:13)	14.31 (3.99) .92 (.56)	12.69 (2.32) − .85 (.96)	
			Interaction: *p* = .264
CCC2 language in context (max score 15)–figurative language (max score 21)			
All participants (n:50) correlation	7.76 (7.12)	16.85 (3.14)	*p *< .001 r = − .560
FwTD (n:11)	.64 (1.29) − 1.00 (.18)	19.38 (.97) − .81 (.28)	Group: *p* = .009
MwTD (n:13)	2.08 (3.57) − .80 (.50)	17.54 (2.76) − .22 (.88)	
FwASD (n:13)	12.92 (4.50) .72 (.63)	16.46 (2.33) .12 (.74)	Gender: *p* = .042
MwASD (n:13)	14.31 (3.99) .92 (.56)	14.00 (3.42) .91 (.74)	
			Interaction: *p* = 757
CCC2 semantics (max score 15)–CELF word association (max score 132)			
All participants (n:50) correlation	5.44 (5.32)	49.06 (12.52)	*p *= .001 r = − .455
FwTD (n:11)	1.00 (1.79) − .83 (.34)	56.62 (8.89) − .60 (.71)	Group: *p* = .008
MwTD (n:13)	1.31 (2.21) − .78 (.42)	51.69 (11.93) − .21 (.95)	
FwASD (n:13)	10.23 (5.10) .90 (.96)	48.69 (13.44) .03 (1.07)	Gender: *p* = .028
MwASD (n:13)	8.54 (3.20) .58 (.60)	39.23 (9.52) .78 (.76)	
			Interaction: *p* = .141
CCC2 syntax (max score 15)–CELF recalling sentences (max score 95)			
All participants (n:50) correlation	2.86 (4.204)	69.00 (12.71)	*p *< .001 r = − .495
FwTD (n:11)	.00 (.00) − .68 (.00)	72.23 (12.71) − .25 (1.00)	Group: *p* = .063
MwTD (n:13)	.46 (.97) − .57 (.23)	74.23 (10.83) − .41 (.85)	
FwASD (n:13)	6.23 (5.20) .80 (1.24)	68.85 (10.13) .01 (.80)	Gender: *p* = .103
MwASD (n:13)	4.31 (3.99) .34 (.95)	60.69 (13.76) .65 (1.09)	
			Interaction: *p* = .017

*Correlations* There were significant correlations between the PRS and DAs (range *p* < .001 to .007), as well as between the CCC-2 and the DA (range *p* .001–.001).

*PRS Total/Pragmatic Measures* Observationally, FwASD and MwASD scored as having more pragmatic difficulties according to clinician observations compared with either direct assessment. FwASD scored more on PRS total (Mean = 11.38, SS = .20) than on DA of bridging coherence (Mean = 14.62, SS = .05) and figurative language (Mean = 16.46, SS = .12). MwASD scored more on PRS total (Mean = 21.69, SS = 1.21) than on DA of bridging coherence (Mean = 12.69, SS = .85) and figurative language (Mean = 14.00, SS = .91). This was significant on the bridging coherence comparison for group [*F*(1, 48) = 53.13, *p* < .001, *ŋ*^*2*^ = .525[, gender [*F*(1, 48) = 11.328, *p* = .002, *ŋ*^*2*^ = .191[ and interaction [*F*(1, 48) = 5.627, *p* = .022, *ŋ*^*2*^ = .105]. But it was not significant for group [*F*(1, 48) = 1.991, *p* = .165, *ŋ*^*2*^ = .040], gender [*F*(1, 48) = 228, *p* = .635, *ŋ*^*2*^ = .005], nor interaction [*F*(1, 48) = 1.791, *p* = .187, *ŋ*^*2*^ = .036] on the figurative language task.

*PRS speech and language/speech and language measures*: Only MwASD were rated by clinicians to have more difficulties with language skills (Mean = 2.85, SS = 1.17) than were identified in the DA of receptive vocabulary (BPVS) (Mean = 104.69, SS = .38). Differences were significant for gender [*F*(1, 48) = 5.915, *p* = .019, *ŋ*^*2*^ = .110], but not group [*F*(1, 48) = 4.005, *p* = .051, *ŋ*^*2*^ = .077] nor interaction [*F*(1, 48) = .985, *p* = .326, *ŋ*^*2*^ = .020]. Observationally, FwASD (Mean = 1.08, SS = .03) and MwASD (Mean = 2.85, SS = 1.17) were both rated by clinicians as having more difficulties with language skills than were evidenced from the DA of recalling sentences (CELF): FwASD (Mean = 68.85, SS = .01) and MwASD (Mean = 60.69, SS.65). However, this did not reach significance for group [*F*(1, 48) = 3.423, *p* = .070, *ŋ*^*2*^ = .067], gender [*F*(1, 48) = 1.545, *p* = .220, *ŋ*^*2*^ = .031] nor interaction [*F*(1, 48) = .268, *p* = .607, *ŋ*^*2*^ = .006].

*CCC2 Language in Context/Pragmatic Measures* FwASD and MwASD scored as having more pragmatic difficulties using parental measures than they did through direct assessment of inference. Parents of FwASD scored more difficulties for the use of language in context (Mean = 12.92, SS = .72) than were identified by DA of bridging coherence (Mean = 14.62, SS = .05) and figurative language (Mean = 16.46, SS = .12). Parents of MwASD scored more difficulties for the use of language in context (Mean = 14.31, SS = .92) than were identified by DA of bridging coherence (Mean = 12.69, SS = .85) and figurative language (Mean = 14.00, SS = .91). This was significant on the bridging coherence comparison for group [*F*(1, 46) = 10.467, *p* = .002, *ŋ*^*2*^ = .185], but not gender [*F*(1, 46) = 1.529, *p* = .223, *ŋ*^*2*^ = .032] nor interaction [*F*(1, 46) = 1.279, *p* = .264, *ŋ*^*2*^ = .027]. It was significant on the figurative language comparison for group [*F*(1, 46) = 7.386, *p* = .009, *ŋ*^*2*^ = .138] and gender [*F*(1, 46) = 4.377, *p* = .042, *ŋ*^*2*^ = .087] but not interaction [*F*(1, 46) = .097, *p* = .757, *ŋ*^*2*^ = .002].

*CCC2 Versus DA (Semantic and Syntactic Measures)* Only FwASD were rated by parents to have more difficulties with semantic skills (Mean = 10.23, SS = .90) than were identified in the DA of semantics (CELF-WA) (Mean = 104.69, SS = .38). Differences were significant for gender [*F*(1, 46) = 5.165, *p* = .028, *ŋ*^*2*^ = .101], and group [*F*(1, 48) = 5.915, *p* = .019, *ŋ*^*2*^ = .110] but not interaction [*F*(1, 46) = 2.241, *p* = .141, *ŋ*^*2*^ = .046]. Only FwASD were rated by parents to have more difficulties with syntactic skills (Mean = 6.23, SS = .80) than were identified in the direct assessment of recalling sentences (CELF-RS) (Mean = 68.85, SS = .01). Differences were not significant for gender [*F*(1, 46) = 2.772, *p* = .103, *ŋ*^*2*^ = .057] nor group [*F*(1, 46) = 3.622, *p* = .063, *ŋ*^*2*^ = .073] but there was a significant interaction [*F*(1, 46) = 6.097, *p* = .017, *ŋ*^*2*^ = .117].

### Summary

Data demonstrated overall correlation between clinician scores and DAs (moderate and small) and between parent scores and DAs (moderate). However, diagnostic group, gender and/or interaction had a significant effect on the difference between these measures. Typically pragmatic measures (bridging coherence and figurative language) showed fewer difficulties for the ASD group when compared with ratings by clinicians or parents. This reached significance when comparing the PRS and CCC-2 to the bridging coherence task, and comparing the CCC-2 to the figurative language task. The pattern of outcomes, when comparing DA language tasks to parent and teacher scores, was more variable. Clinicians rated MwASD (only) as having significantly more functional language difficulties than those identified with DA of vocabulary and recalling sentences. Parents rated FwASD (only) as having significantly more functional language difficulties when compared with DA of semantic and syntactic ability.

## Discussion

This study provides new information contributing to our understanding of the female phenotype in autism. It demonstrates gender difference in observable pragmatic skills and social/emotional behaviours. It also provides detail on the patterns of scoring differences evident across pragmatic/language assessment measures. This provides important information on how different reporters perceive difficulties for FwASD, with implications for clinical and research data collection. Key findings are explored below.

1. Females with ASD Demonstrate Differences in Their Observable Pragmatic, Language and Social–Emotional Behaviours Compared with Males with ASD and Typically Developing Controls

Perhaps the most important finding from this study is further evidence of difference between females and males with ASD. As predicted, the PRS showed group and gender differences on total scores as well as summative (overall and impact) and composite sub-scores (discourse management, speech and language, non verbal behaviours). This supports existing research suggesting FwASD have better skills in conversational reciprocity (Head et al. 2014), language and pragmatics (Conlon et al. 2018; Sturrock et al. 2019) and non-verbal communication (Holtmann et al. 2007; Park et al. 2012) compared with MwASD. It also builds on preliminary investigations into gender differences identified using the PRS (Dillon et al. 2018). By providing gender-normative data our study shows that FwASD were positioned in the middle of a performance slope, scoring better than MwASD but worse than FwTD. This mirrors findings on measures of language (Sturrock et al. 2019), emotional reciprocity (Head et al. 2014) and pretend play (Knickmeyer et al. 2008). The PRS is a measure of subtle symptomatology associated with ASD (Landa 2011, 2013), potentially making it a good tool to identify subtle behavioural differences exhibited between females and males. The speech acts sub-score failed to show group or gender difference, which may indicate a ceiling effect or a strength for higher IQ children in all groups.

Parents also identified a heightened level of emotional difficulty for FwASD, who scored more difficulties than all other groups on this composite score. Teachers showed the same trend, although this did not reach significance. Increased difficulties with social-emotional behaviours have been reported in the wider literature (Horovitz et al. 2011; Solomon et al. 2012; Worley and Matson 2011). Overall parents, teachers and children reported difficulties mostly following the aforementioned slope pattern. Differences reached significance for group but rarely gender. However, parents were more likely than any other reporters to identify FwASD as the lowest performing group. Both parents and teachers found FwASD to have greatest difficulty on the internalising sub-score (SDQ) compared with other participant groups. This difference may be predicted from the wider literature (Giarelli et al. 2010) and lack of significance in this study may be due to the small sample size.

2. The Children and Parents of Children with ASD do not Experience a Gender Difference in Pragmatic, Language and Social Behaviours

It is notable that children in this study (ASD in middle-childhood) were able to identify lower proficiency in their pragmatic, language and social domains than their TD peers. It points to an early awareness of feeling different, often reported in the literature (Holliday Willey 2015, Sedgewick et al. 2016). This potentially feeds into reported feelings of loneliness and poorer well-being (Mazurek 2013). Parents also identified more difficulties compared with parents of TD peers, and this was consistent for males and females. To some extent this is consistent with the literature. Despite evidence that FwASD objectively present with a more subtle symptomatology than males (Knickmeyer et al. 2008; Mandy et al. 2012; Park et al. 2012), other factors have been shown to impact on parental and self-reports. Previous research shows FwASD and their parents both report more difficulties than clinical scores would predict (Holtmann et al. 2007, and Lai et al. 2011), which may be driven by higher societal expectations for this group. Additionally, gender-normative data support the notion of FwASD underperforming relative to TD peers (Head et al. 2014; Knickmeyer et al. 2008; Sturrock et al. 2019). It is likely that the experiential perspective of parents and the individual will be based on comparisons of the individual to gender-matched peers and not to other children with ASD. In this respect, FwASD will be equally disadvantaged compared with their TD peers, as MwASD will be with theirs. This will impact on perceived severity of difficulties.

3. Parents are more Likely to Identify Difficulties than Clinicians on Language and Pragmatic Assessments and are more Likely than Teachers to do so on Behavioural Measures for the Same Child

Overall, parent/clinician and parent/teacher reports were well correlated. However, differences according to gender and diagnostic group were evidenced throughout. Clinical observations on the PRS scored fewer difficulties than parent ratings on the CCC-2. Teacher reports identified fewer difficulties than parents on the SDQ. In both cases there was a greater effect for FwASD, potentially driven by parents greater likelihood to rate FwASD with worse difficulties than MwASD. This discrepancy could result from experiential differences impacting on reporting of difficulties by parents (previously discussed). It could also result from behavioural differences associated with location/situation (Posserud et al. 2006), i.e. the home versus school/test environment. There may be a potentially inflated effect for FwASD who are more likely to camouflage undesired behaviours in social situations (Bargiela et al. 2016). Differences between reported behaviours of boys and girls across the school/home environment have been reported in the literature (Mandy et al. 2012) and could explain differences on this measure in our study.

4. Males with ASD are more Likely to Under-Identify Difficulties with Pragmatic, Language and Social Behaviours than Females with ASD

Child self-reporting in ASD is relatively un-explored and this study provides new data on gender-differences in this respect. In particular, MwASD were more likely to under-identify difficulties in comparison to both parents and clinicians, which supports the notion that ASD groups have poorer self-awareness than TD controls (Huang et al. 2017). In our study FwASD were also likely to under-identify difficulties compared with parents, which implies underlying difficulties similar to male peers. However, they were likely to over-identify difficulties when compared with clinical observations. This reflects findings by Lai et al. (2011), in which FwASD reported more difficulties compared with clinical ratings than males, either due to better or more critical self-awareness. Better self-awareness (Elmose 2016) may be driven by greater interest in relationships (Head et al. 2014) and more person-centred discussions within peer relations (Sedgewick et al. 2016). More critical self-reflection may reflect heightened expectation of social competency for FwASD and real performance differences noted between this group and TD females (previously noted). Differences in scores are also relative to each other. Therefore, parents rating higher or clinicians rating lower levels of difficulty may be driving these trends. Overall FwASD showed a pattern distinct from either MwASD or TD groups. Interestingly, TD children always rated more difficulties for themselves than either parents or clinicians. This is a novel finding and its interpretation is not clear. One possibility is that children with TD are more sensitive to the pragmatic, language and social difficulties they experience than either their parents or ASD children. This may be a supportive factor in developing social competencies. More research in this area would elucidate findings.

5. Direct Assessment may Provide Useful Information About Performance on Language and Pragmatics in Optimal Conditions, but may Underestimate Difficulties Observed in Functional Settings, Especially for Pragmatic Abilities

As predicted, DAs consistently showed better results for the ASD group when compared with functional reports. Pragmatic tasks (bridging coherence and figurative language), in particular, showed significance on three of the four comparisons (with CCC-2 and PRS). It is important to note that DAs were primarily assessing inferred pragmatic ability (i.e. interpreting inferred information), while functional report tends to focus on observable difficulties (i.e. ‘does not understand jokes’ and ‘provides too much detail’). However, overall scores were well correlated with greatest differences between measures associated with diagnosis. Scores on language measures were more complex to interpret. MwASD had more difficulties with speech and language according to clinical observations than were identified using the BPVS. However, parents rated FwASD worse on semantic and syntax composite scores compared with DAs of semantic or sentence recall. In previous work MwASD and FwASD performed equitably on scores of structural language measures (Sturrock et al. 2019), which may indicate differences in reporter perceptions. Null results may also be due to the relatively small sample size. The overall pattern suggests DAs underestimate difficulties for the ASD group compared with functional reports, which may be due to DAs providing an optimum environment, with isolated tasks and fewer distractions (Frith and Happé 1994) and time for applying preferred logical processing methods (Baron-Cohen 2002, Lai et al. 2012). Pragmatic tasks may be particularly affected by the test setting, as predicted by Adams (2002), with additional demands of interpreting context being fundamentally changed.

### Limitations and Future Research

This is a relatively small study with small effect sizes raising the potential for type II errors. Findings nearing significance might have reached significance given a larger data set. This study also entailed a secondary analysis of subsections (e.g. language in context) of the primary data set (e.g. CCC-2). Although these did constitute separate variables, this approach may raise the possibility of type I errors. Our primary findings are given more weight in the discussions and implications, and our secondary analyses of subsections are reported, with the hope that they will inform future research in this area. We believe every caution has been taken to present the results meaningfully, but, of course, significance levels should be interpreted with caution. Overall, the findings showed a great deal of consistency between each other and with the wider literature, but a larger scale study, should be undertaken to validate the results.

The CC-SR was presented to children younger than the 10 year cut off. Age was well-matched between groups and so likely affected all groups similarly. Questions were also scaffolded by the clinician to facilitate self-report. However, it is possible that other factors may disproportionately impact on younger ASD children thereby affecting results. Several comparisons were made across different measures, with items carefully selected to represent the same features, but, other items could have been selected for comparison, potentially generating different results. It is hoped this study will contribute to a wider discussion about the relative strengths and weaknesses of data collection methods and materials.

Report measures and PRS observations allow for the potential of subjectivity in responses. Exploring those biases, by comparing assessment measures, was part of the study aims. When conducting the PRS it was not possible to blind raters to the gender (or effectively the diagnosis) of the children being assessed. This is a recognised weakness in data collection using observational measures of behaviour and a known weakness in our study design. In this respect we are reliant to some extent on clinical and researcher integrity. However, results were well correlated between the measures used in this study and differences were in line with expectations from the wider literature.

FwASD group were selected to represent the group least likely to receive diagnosis (those in middle childhood with a higher IQ). However, our group did have a diagnosis and so may still over represent females with a more male-type profile. This may also explain our lack of gender difference in the autism severity ratings using the ASSQ. However, it is also worth noting that, as a parental measure, this may be subject to the same biases as noted in the body of our work. In our experience, the study group did succeed in reflecting FwASD often missed from diagnosis. Additionally, results demonstrated gender differences in terms of their pragmatics, language and social profile and perspectives of clinicians, parents, teachers and the individual. We argue that this study provides novel preliminary data and points to new areas of investigation for the future.

### Implications and Conclusions

It is thought that the key findings of this research will contribute to our understanding of the female phenotype of autism. Building a clear profile for FwASD could improve recognition, referral and accurate diagnosis of this group. In addition, by providing gender-normative data, we show the relative strengths and weakness experienced by this group in comparison to TD peers. Understanding disadvantages here could lead to targeted therapy for this group, focusing on better social function, improving chances of friendship-building and ultimately well-being. Additionally, this paper draws wider conclusions about the use of observational, report and direct assessment. It considers their relative use in developing a profile of abilities, and demonstrates potential biases. With all methods showing some limitations, it is the recommendation of this paper that clinicians should provide holistic assessment; include a range of measures and be aware of those potential biases. The *truth* about an individual’s academic ability in optimal settings, versus functional communicative settings and behavioural responses in the home versus the school, are all different *truths* about the same individual. Research should also be aware of potential influencing factors impacting on results from clinician, parent, teacher or self-reports and direct assessments, and consider this in data interpretation.

## Electronic supplementary material

Below is the link to the electronic supplementary material.

Supplementary material 1 (DOCX 220 kb)
