# Supplementary material for: Observational and Reported Measures of Language and Pragmatics in Young People with Autism: A Comparison of Respondent Data and Gender Profiles
Source: J Autism Dev Disord. 2019 Nov 22;50(3):812–30. doi: 10.1007/s10803-019-04288-3 (PMC7010622; doi:10.1007/s10803-019-04288-3)
Supplement: Supplementary file 1 — Supplementary material 1 (DOCX 220 kb) [file 10803_2019_4288_MOESM1_ESM.docx]

**Appendix**

*Appendix 1: Local Coherence Inference task*

Original material by Joliffe and Baron-Cohen (1999). The child reads a short story which purposely omits an overt bridging reference between an initiating event and a consequence. The child is asked to correctly identify the missing information from a choice of three, all of which could be appropriate but one constitutes the best fit. Responses were scored correct/incorrect and timed between the end of reading the story and selection of an option. This test measure was selected due to previous evidence of demonstrating difference between adults with HFASD and TD. Original material was modified to suit the younger participants and in the following ways:

- 18 test items were derived from the six presented in the original study design
- New test items were matched for sentence length
- All vocabulary choices, language complexity and topics were tested for age appropriacy with a TD middle childhood pilot study.
- Test items were found to be appropriate for the age group and there were no floor or ceiling effects in results.
- Minor adaptations were made following feedback.

*
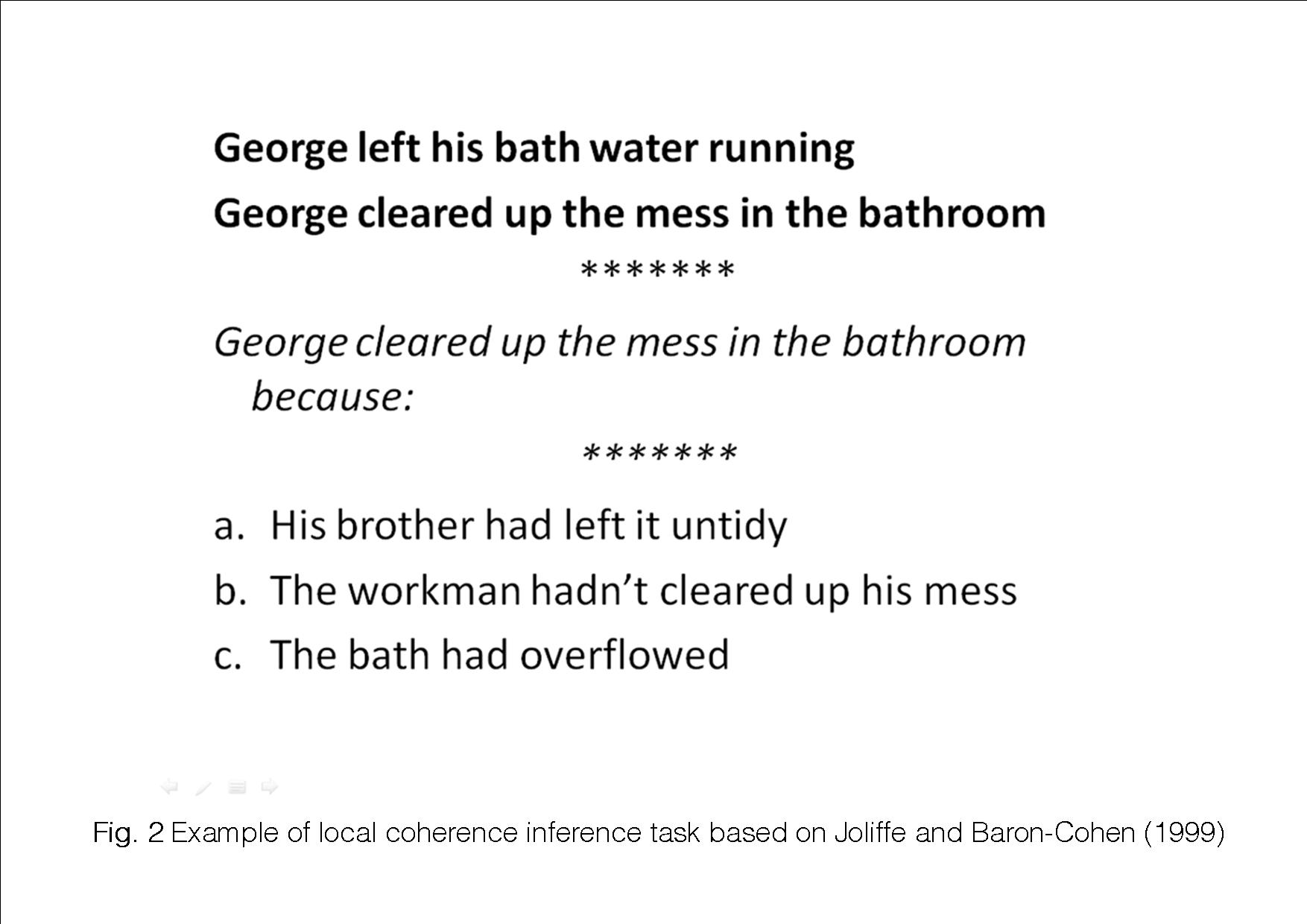
*

*Appendix 2: Figurative language task*

Original material by (MacKay & Shaw, 2004), the child is asked to describe the true meaning of the figurative language and what intention the speaker had for using a non-literal phrase. One point was given for each correct response in line with original study scoring criteria. This measure was chosen because of its previous success evidencing difference between children with HFASD and TD. Test items cover 3 examples of each; irony, hyperbole, metonym, indirect comment, rhetorical question, understatement and metaphor. Metaphor was added in our adapted version in order to provide a thorough coverage of figurative language types. It was adapted in the following ways:

- Number of test items for figurative the original list of language forms( irony, hyperbole, metonymy, indirect comment, rhetorical question, understatement) were reduced from 10 repetitions of each item, to 3 repetitions of each item. Resulting in 18 test items
- Three examples of metaphor were also added yielding 21 test items.
- Metaphor examples were matched to original material in terms of story length and word appropriacy for age group
- Materials were presented on a Power Point presentation rather than pieces of paper
- Images were taken from the internet and identified as successfully representing the true meaning of the figurative language sample
- All adaptations were peer reviewed to ascertain appropriacy for the participant population

| **Figurative language type & no.** | **Sample sentence** |
| --- | --- |
| Hyperbole | You have millions of CDs (lots of) |
| Indirect Requests | It’s very noisy out there (be quiet) |
| Metonymy | Quieten down sandpit (children in the sandpit) |
| Irony  Rhetorical Questions | Great singing mum (dreadful singing)  Are you trying to get yourself killed? |
| Understatement | Just a few things (lots of shopping) |
| Metaphor | My teacher is a dragon (horrible) |

*
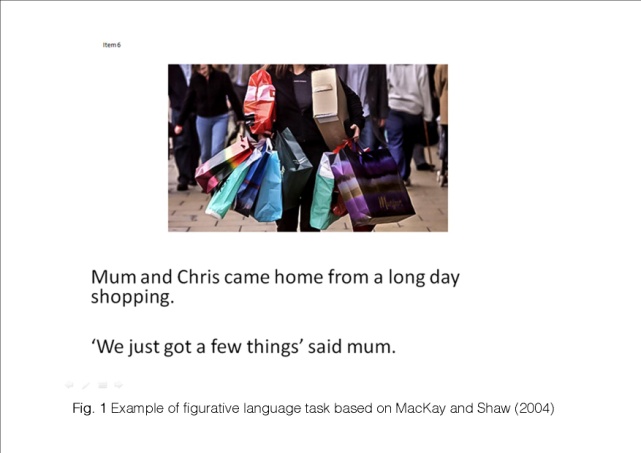
*

*Appendix 3: Table 6: Pragmatic Rating Scale (PRS) secondary/subsection analysis*

| Table 6: Descriptive statistics for total and composite scores on the Pragmatic Rating Scale 2x groups by 2xgender ANCOVA; covariant PIQ | | | | | | |
| --- | --- | --- | --- | --- | --- | --- |
|  | ASD  (n = 26)  Mean (SD) | TD  (n = 25)  Mean (SD) | Gender overall  Mean (SD) | Significance  on measure | | |
|  | Raw  Score | Raw  Score |  | Group | Gender | Interaction |
| Overall score  (max score 2) |  |  |  | *p* = <.001 | *p* = .049 | *p* = .389 |
| Female | 1.23  (.439) | .25  (.452) | .76  (.663) |  |  |  |
| Male | 1.62  (.506) | .38  (.506) | 1.00  (.800) |  |  |  |
| Group overall | 1.42  (.504) | .32  (.476) | .88  (.739) |  |  |  |
| Impact score  (max score 3) |  |  |  | *p* = <.001 | *p* = .003 | *p* = .003 |
| Female | .92  (1.32) | .00  (.00) | .48  (1.05) |  |  |  |
| Male | 2.28  (1.04) | .00  (.00) | 1.19  (1.42) |  |  |  |
| Group overall | 1.65  (1.38) | .00  (.00) | .84  (1.29) |  |  |  |
| Speech acts  (max score 4) |  |  |  | *p* = <.001 | *p* = .631 | *p* = .993 |
| Female | .923  (1.12) | .077  (.275) | .500  (.906) |  |  |  |
| Male | .846  (.689) | .000  (.000) | .423  (6.43) |  |  |  |
| Group overall | .885  (.909) | .038  (.196) | .462  (.779) |  |  |  |
| Theory of Mind  (max score 16) |  |  |  | *p* = <.001 | *p* = .075 | *p* = .159 |
| Female | 3.15  (2.12) | .08  (.29) | 1.68  (2.17) |  |  |  |
| Male | 5.00  (3.37) | .308  (0.63) | 2.65  (3.37) |  |  |  |
| Group overall | 4.08  (2.91) | .20  (.50) | 2.18  (2.86) |  |  |  |
| Discourse Management  (max score 16) |  |  |  | *p* = <.001 | *p* = .002 | *p* = .003 |
| Female | 2.69  (2.90) | .42  (1.16) | 1.60  (2.48) |  |  |  |
| Male | 6.62  (2.93) | .46  (.97) | 3.54  (3.80) |  |  |  |
| Group overall | 4.65  (3.49) | .44  (1.04) | 2.59  (3.34) |  |  |  |
| Speech and Language  (max score 8) |  |  |  | *p* = <.001 | *p* = .004 | *p* = .008 |
| Female | 1.08  (0.76) | .08  (0.29) | .60  (0.76) |  |  |  |
| Male | 2.85  (1.99) | .15  (.38) | 1.50  (1.96) |  |  |  |
| Group overall | 1.96  (1.73) | .12  (.33) | 1.06  (1.55) |  |  |  |
| Supra-segmental  (max score 12) |  |  |  | *p* = .001 | *p* = .098 | *p* = .124 |
| Female | 1.77  (1.92) | .50  (1.24) | 1.16  (1.72) |  |  |  |
| Male | 3.46  (2.57) | .62  (1.39) | 2.04  (2.49) |  |  |  |
| Group overall | 2.62  (2.38) | .56  (1.30) | 1.61  (2.17) |  |  |  |
| Non-Verbal  (max score 12) |  |  |  | *p* = <.001 | *p* = .003 | *p* = .058 |
| Female | 1.92  (1.66) | .58  (1.16) | 1.28  (1.57) |  |  |  |
| Male | 4.23  (1.92) | 1.15  (1.72) | 2.69  (2.38) |  |  |  |
| Group overall | 3.08  (2.12) | .88  (1.48) | 2.00  (2.13) |  |  |  |

*Appendix 4: Table 7: Childhood Communication Checklist – second edition (CCC-2) secondary/subsection analysis*

| Table 7: Descriptive statistics on total and composite scores on the Children’s Communication Checklist: 2xgroup 2x gender ANCOVA; covariant PIQ | | | | | | |
| --- | --- | --- | --- | --- | --- | --- |
|  | ASD  (n = 26)  Mean (SD) | TD  (n = 26)  Mean (SD) | Gender overall  Mean (SD) | Significance  on measure | | |
|  | Raw  Score | Raw  Score |  | Group | Gender | Interaction |
| General communication Composite score |  |  |  | *p* = <.001 | *p* = .597 | *p* = .216 |
| Female | 22.39  (14.28) | 89.92  (12.67) | 56.15  (36.89) |  |  |  |
| Male | 28.08  (12.33) | 86.92  (18.06) | 57.50  (33.61) |  |  |  |
| Group overall | 25.23  (13.39) | 88.42  (15.38) | 56.83  (34.95) |  |  |  |
| Social Deviance composite score |  |  |  | *p* = .016 | *p* = .128 | *p* = .695 |
| Female | -4.54  (11.69) | -.846  (7.95) | -2.69  (9.98) |  |  |  |
| Male | -9.00  (8.88) | -3.46  (6.67) | -6.23  (8.19) |  |  |  |
| Group overall | -6.77  (10.42) | -2.15  (7.31) | -4.46  (9.21) |  |  |  |
| Speech composite score  (max score 21) |  |  |  | *p* = .002 | *p* = .014 | *p* = .251 |
| Female | 6.62  (6.09) | .545  (1.21) | 3.83  (3.83) |  |  |  |
| Male | 3.54  (3.07) | .08  (.28) | 1.81  (2.78) |  |  |  |
| Group overall | 5.08  (4.98) | .29  (.86) | 2.78  (4.34) |  |  |  |
| Syntax composite score  (max score 21) |  |  |  | *p* = <.001 | *p* = .161 | *p* = .279 |
| Female | 6.23  (5.20) | .001  (.00) | 3.38  (4.92) |  |  |  |
| Male | 4.31  (3.99) | .46  (.97) | 2.39  (3.45) |  |  |  |
| Group overall | 5.27  (4.64) | .25  (.74) | 2.86  (4.20) |  |  |  |
| Semantic composite score  (max score 21) |  |  |  | *p* = <.001 | *p* = .178 | *p* = .392 |
| Female | 10.23  (5.10) | 1.00  (1.79) | 6.00  (6.09) |  |  |  |
| Male | 8.54  (3.20) | 1.31  (2.21) | 4.92  (4.57) |  |  |  |
| Group overall | 9.39  (4.26) | 1.17  (1.99) | 5.44  (5.32) |  |  |  |
| Coherence composite score  (max score 21) |  |  |  | *p* = <.001 | *p* = .742 | *p* = .788 |
| Female | 9.77  (5.45) | .73  (1.20) | 5.63  (6.11) |  |  |  |
| Male | 10.46  (3.31) | 1.39  (2.50) | 5.92  (5.48) |  |  |  |
| Group overall | 10.12  (4.43) | 1.08  (1.99) | 5.78  (5.72) |  |  |  |
| Inappropriate initiation score  (max score 21) |  |  |  | *p* = <.001 | *p* = .245 | *p* = .289 |
| Female | 12.23  (4.80) | 1.55  (2.07) | 7.33  (6.59) |  |  |  |
| Male | 14.61  (3.36) | 2.31  (4.17) | 8.46  (7.29) |  |  |  |
| Group overall | 13.42  (4.23) | 1.96  (3.33) | 7.92  (6.92) |  |  |  |
| Stereotypies composite score  (max score 21) |  |  |  | *p* = <.001 | *p* = .891 | *p* = .811 |
| Female | 8.31  (4.57) | .82  (1.60) | 4.88  (5.15) |  |  |  |
| Male | 8.69  (3.90) | .85  (1.82) | 4.77  (4.99) |  |  |  |
| Group overall | 8.50  (4.17) | .83  (1.69) | 4.82  (5.02) |  |  |  |
| Use of context composite score  (max score 21) |  |  |  | *p* = <.001 | *p* = .268 | *p* = .865 |
| Female | 12.92  (4.50) | .64  (1.29) | 7.29  (7.10) |  |  |  |
| Male | 14.31  (3.99) | 2.08  (3.57) | 8.19  (7.26) |  |  |  |
| Group overall | 13.62  (4.22) | 1.42  (2.81) | 7.76  (7.12) |  |  |  |
| Non-verbal composite score  (max score 21) |  |  |  | *p* = <.001 | *p* = .475 | *p* = .363 |
| Female | 14.08  (3.62) | .55  (1.21) | 7.88  (7.41) |  |  |  |
| Male | 12.92  (3.99) | 1.23  (2.01) | 7.08  (6.72) |  |  |  |
| Group overall | 13.50  (3.78) | .92  (1.69) | 7.46  (7.00) |  |  |  |
| Social composite score  (max score 21) |  |  |  | *p* = <.001 | *p* = .948 | *p* = .705 |
| Female | 11.69  (3.52) | 1.36  (2.50) | 6.96  (6.07) |  |  |  |
| Male | 12.08  (4.84) | 1.31  (2.18) | 6.70  (6.61) |  |  |  |
| Group overall | 11.86  (4.15) | 1.33  (2.28) | 6.82  (6.29) |  |  |  |
| Interests composite score  (max score 21) |  |  |  | *p* = <.001 | *p* = .220 | *p* = .523 |
| Female | 13.85  (2.97) | 2.91  (2.58) | 8.83  (6.20) |  |  |  |
| Male | 15.46  (2.37) | 3.62  (3.59) | 9.54  (6.74) |  |  |  |
| Group overall | 14.65  (2.76) | 3.29  (3.11) | 9.20  (6.43) |  |  |  |

*Appendix 5: Table 8: Communication Checklist – Self report (CC-SR) secondary/subsection analysis*

| Table 8: Descriptive statistics on total and composite scores on the Communication Checklist – Self Report: 2x group 2x gender ANCOVA; covariant PIQ | | | | | | |
| --- | --- | --- | --- | --- | --- | --- |
|  | ASD  (n = 25)  Mean (SD) | TD  (n = 26)  Mean (SD) | Gender overall  Mean (SD) | Significance  on measure | | |
|  | Raw  Score | Raw  Score |  | Group | Gender | Interaction |
| Language structure composite score  (max score 63) |  |  |  | *p* = .084 | *p* = .505 | *p* = .941 |
| Female | 13.00  (10.05) | 8.46  (5.81) | 10.64  (8.28) |  |  |  |
| Male | 14.78  (8.34) | 9.85  (6.81) | 12.31  (7.87) |  |  |  |
| Group overall | 13.92  (9.05) | 9.15  (6.24) | 11.49  (8.39) |  |  |  |
| Pragmatic skills composite score  (max score 66) |  |  |  | *p* = .019 | *p* = .800 | *p* = .858 |
| Female | 18.33  (10.08) | 11.00  (9.27) | 14.52  (10.17) |  |  |  |
| Male | 18.58  (9.65) | 12.15  (7.64) | 15.37  (9.13) |  |  |  |
| Group overall | 18.46  (9.65) | 11.58  (8.34) | 14.95  (9.57) |  |  |  |
| Social engagement composite score  (max score 81) |  |  |  | *p* = .008 | *p* = .173 | *p* = .414 |
| Female | 30.82  (9.80) | 18.31  (6.99) | 24.04  (10.39) |  |  |  |
| Male | 32.82  (11.24) | 24.69  (12.48) | 28.76  (12.35) |  |  |  |
| Group overall | 31.90  (10.43) | 21.50  (10.43) | 26.49  (11.58) |  |  |  |

*Appendix 6: Table 9: Strengths and Difficulties Questionnaire - Parent report (SDQ-P) secondary/subsection analysis*

| Table 9: Descriptive statistics on total and composite scores using the Strengths and Difficulties Questionnaire (parent-rater) comparison: 2x group 2x gender ANCOVA; covariant PIQ | | | | | | |
| --- | --- | --- | --- | --- | --- | --- |
|  | ASD  (n = 26)  Mean (SD) | TD  (n = 26)  Mean (SD) | Gender overall  Mean (SD) | Significance  on measure | | |
|  | Raw  Score | Raw  Score |  | Group | Gender | Interaction |
| Pro-social score  (max score10) |  |  |  | *p* = <.001 | *p* = .866 | *p* = .703 |
| Female | 4.27  (2.09) | 9.15  (1.14) | 6.71  (2.99) |  |  |  |
| Male | 4.38  (2.96) | 8.85  (1.07) | 6.62  (3.15) |  |  |  |
| Group overall | 4.33  (2.51) | 9.00  (1.10) | 6.66  (3.04) |  |  |  |
| Peer problems  (max score 10) |  |  |  | *p* = <.001 | *p* = .445 | *p* = .793 |
| Female | 5.23  (2.65) | .92  (1.38) | 3.08  (3.02) |  |  |  |
| Male | 5.85  (2.27) | 1.23  (1.69) | 3.54  (3.06) |  |  |  |
| Group overall | 5.54  (2.44) | 1.08  (1.52) | 3.31  (3.02) |  |  |  |
| Emotion scores  (max score 10) |  |  |  | *p* = <.001 | *p* = .010 | *p* = .151 |
| Female | 8.38  (1.61) | 1.31  (1.75) | 4.85  (3.97) |  |  |  |
| Male | 6.31  (2.53) | .69  (.86) | 3.50  (3.41) |  |  |  |
| Group overall | 7.35  (2.33) | 1.00  (1.39) | 4.17  (3.73) |  |  |  |
| Conduct score  (max score 10) |  |  |  | *p* = <.001 | *p* = .814 | *p* = .216 |
| Female | 4.62  (2.66) | .54  (1.13) | 2.58  (2.89) |  |  |  |
| Male | 3.92  (1.89) | 1.08  (1.32) | 2.50  (2.16) |  |  |  |
| Group overall | 4.27  (2.29) | .81  (1.23) | 2.54  (2.52) |  |  |  |
| Hyperactivity score  (max score 10) |  |  |  | *p* = <.001 | *p* = .876 | *p* = .806 |
| Female | 7.23  (2.92) | 1.92  (1.71) | 4.58  (3.58) |  |  |  |
| Male | 7.23  (2.35) | 2.23  (2.56) | 4.73  (3.51) |  |  |  |
| Group overall | 7.23  (2.60) | 2.08  (2.13) | 4.65  (3.51) |  |  |  |
| Internalising behaviours composite score  (max score 20) |  |  |  | *p* = <.001 | *p* = .285 | *p* = .391 |
| Female | 13.62  (3.07) | 2.17  (2.86) | 8.12  (6.52) |  |  |  |
| Male | 12.15  (2.91) | 2.00  (1.83) | 7.08  (5.70) |  |  |  |
| Group overall | 12.88  (3.02) | 2.08  (2.33) | 7.59  (6.08) |  |  |  |
| Externalising behaviours composite score  (max score 20) |  |  |  | *p* = <.001 | *p* = .762 | *p* = .178 |
| Female | 12.62  (3.10) | 2.42  (2.61) | 7.72  (5.91) |  |  |  |
| Male | 11.15  (3.81) | 3.38  (3.71) | 7.27  (5.41) |  |  |  |
| Group overall | 11.88  (3.48) | 2.92  (3.20) | 7.49  (5.61) |  |  |  |
| Impact total score  (max score 10) |  |  |  | *p* = <.001 | *p* = .375 | *p* = .079 |
| Female | 5.75  (2.18) | .69  (1.38) | 3.12  (3.13) |  |  |  |
| Male | 7.08  (2.60) | .31  (.63) | 3.69  (3.92) |  |  |  |
| Group overall | 6.44  (2.45) | .50  (1.07) | 3.41  (3.53) |  |  |  |

*Appendix 7: Table 10: Strengths and Difficulties Questionnaire - Teacher Report (SDQ-T) secondary/subsection analysis*

| Table 10: Descriptive statistics on total and composite scores using the Strengths and Difficulties Questionnaire (teacher-rater): 2x groups by 2x gender ANCOVA; covariant PIQ | | | | | | |
| --- | --- | --- | --- | --- | --- | --- |
|  | ASD  (n = 22)  Mean (SD) | TD  (n = 25)  Mean (SD) | Gender overall  Mean (SD) | Significance  on measure | | |
|  | Raw  Score | Raw  Score |  | Group | Gender | Interaction |
| Pro-social score  (max score 10) |  |  |  | *p* = <.001 | *p* = .289 | *p* = .699 |
| Female | 6.09  (1.97) | 9.13  (1.48) | 7.67  (2.29) |  |  |  |
| Male | 5.18  (3.31) | 8.69  (1.49) | 7.08  (3.02) |  |  |  |
| Group overall | 5.64  (2.70) | 8.90  (1.47) | 7.37  (2.68) |  |  |  |
| Peer score  (max score 10) |  |  |  | *p* = .001 | *p* = .115 | *p* = .057 |
| Female | 1.59  (2.15) | .58  (.90) | 1.07  (1.67) |  |  |  |
| Male | 3.09  (1.76) | .46  (.97) | 1.67  (1.90) |  |  |  |
| Group overall | 2.34  (2.07) | .52  (.92) | 1.37  (1.80) |  |  |  |
| Emotion score  (max score 10) |  |  |  | *p* = <.001 | *p* = .217 | *p* = .432 |
| Female | 5.00  (1.90) | 1.00  (1.76) | 2.91  (2.71) |  |  |  |
| Male | 3.19  (2.12) | .77  (1.48) | 2.21  (2.38) |  |  |  |
| Group overall | 4.45  (2.04) | .88  (1.59) | 2.55  (2.54) |  |  |  |
| Conduct score  (max score 10) |  |  |  | *p* = .096 | *p* = .119 | *p* = .618 |
| Female | 1.27  (1.79) | .25  (.45) | .74  (1.36) |  |  |  |
| Male | 2.18  (2.27) | .77  (1.69) | 1.42  (2.06) |  |  |  |
| Group overall | 1.73  (2.05) | .52  (1.26) | 1.09  (1.77) |  |  |  |
| Hyperactivity score  (max score 10) |  |  |  | *p* = <.001 | *p* = .060 | *p* = .214 |
| Female | 3.64  (3.30) | .92  (1.17) | 2.22  (2.75) |  |  |  |
| Male | 6.00  (3.44) | 1.46  (2.37) | 3.54  (3.66) |  |  |  |
| Group overall | 4.82  (3.50) | 1.20  (1.87) | 2.89  (3.28) |  |  |  |
| Internalising composite score  (max score 20) |  |  |  | *p* = <.001 | *p* = .475 | *p* = .926 |
| Female | 7.56  (3.47) | 1.89  (2.67) | 4.72  (4.18) |  |  |  |
| Male | 6.78  (2.44) | 1.15  (1.94) | 3.82  (3.59) |  |  |  |
| Group overall | 7.17  (2.94) | 1.50  (2.28) | 4.26  (3.86) |  |  |  |
| Externalising composite score  (max score 20) |  |  |  | *p* = .017 | *p* = .037 | *p* = .357 |
| Female | 4.22  (4.49) | 1.11  (1.54) | 2.67  (3.63) |  |  |  |
| Male | 7.67  (4.95) | 2.50  (4.30) | 4.95  (5.21) |  |  |  |
| Group overall | 5.94  (4.92) | 1.84  (3.29) | 3.84  (4.60) |  |  |  |
| Total impact score  (max score 10) |  |  |  | *p* = .012 | *p* = .549 | *p* = .276 |
| Female | 1.20  (1.55) | .18  (.603) | .67  (1.24) |  |  |  |
| Male | 1.82  (1.88) | .00  (.00) | .91  (1.60) |  |  |  |
| Group overall | 1.52  (1.72) | .09  (.43) | .79  (1.42) |  |  |  |
